# A Meta-Analysis Comparing Postoperative Complications and Outcomes of Femtosecond Laser-Assisted Cataract Surgery versus Conventional Phacoemulsification for Cataract

**DOI:** 10.1155/2017/3849152

**Published:** 2017-04-30

**Authors:** Zi Ye, Zhaohui Li, Shouzhi He

**Affiliations:** Department of Ophthalmology, The PLA General Hospital, 28 Fuxing Road, Beijing 100853, China

## Abstract

*Objective*. This meta-analysis aimed to compare the outcomes and postoperative complications between femtosecond laser-assisted cataract surgery (FLACS) and conventional phacoemulsification cataract surgery (CPCS). *Methods*. Bibliographic databases, including PubMed, Embase, and Cochrane library, were systematically searched for references on or before September 2015 regarding the outcomes and complications by FLACS or CPCS. Data on corneal endothelial cell loss, uncorrected distance visual acuity (UDVA), corrected distance visual acuity (CDVA), refractive outcomes, and postoperative complications were retrieved. *Results*. A total of 9 trials were included in this analysis. Refractive outcomes (MD = −0.21, 95% CI: −0.39~0.03, *P* = 0.02) were significantly improved after FLACS. Although corneal endothelial cell loss was not significantly reduced after FLACS, there was a trend towards lower corneal endothelial cell loss (mean difference (MD) = 197.82, 95% confidence interval (CI): 2.66~392.97, *P* = 0.05) after FLACS. There was no significant difference in UDVA (MD = −0.01, 95% CI: −0.13~0.10, *P* = 0.80) or CDVA (MD = −0.03, 95% CI: 0.07~0.00, *P* = 0.09) between the two surgeries. Elevated intraocular pressure and macular edema were most commonly developed complications after cataract surgery, and the incidence of these complications associated with the two surgeries was similar. *Conclusion*. Compared with CPCS, FLACS might achieve higher refractive stability and corneal endothelial cell count. Nevertheless, further study is needed to validate our findings.

## 1. Introduction

Cataract is responsible for 48% of worldwide blindness, especially in developed countries [1, 2]. Conventional phacoemulsification cataract surgery (CPCS) is the most common surgical treatment for cataract. CPCS is generally effective for cataract but may cause a few complications such as elevated intraocular pressure and macular edema probably due to the heat generated by ultrasound during the procedure [3]. Femtosecond laser-assisted cataract surgery (FLACS), a new technology that was firstly introduced in 2008 [4], has shown promising treatment outcomes. To date, many studies have attempted to compare the outcome and complications of FLACS and CPCS. Some studies have shown better visual acuity recovery and lower endothelial cell loss after FLACS when compared with CPCS [5, 6], whereas others have detected no significant difference between the two technologies [7, 8]. We herein performed this systematic study in order to evaluate the treatment efficacy and complications of FLACS and CPCS, including visual recovery, corneal cell integrity, and functionality in an aim to provide guidance for clinical practice.

## 2. Material and Methods

### 2.1. Literature Search

Bibliographic databases, including PubMed, Embase, and Cochrane library, were systematically searched to identify eligible studies until September 2015. The search key words were used including “femtosecond” AND “phaco OR phacoemulsification OR phakoemulsification” AND “cataract.”

### 2.2. Selection Criteria

Studies meeting the following criteria were included in the meta-analysis: (1) studies designed as prospective studies; (2) cataract patients were divided into FLACS and CPCS groups; (3) at least one of the following outcomes was reported: corneal endothelial cell counts, central corneal thickness, uncorrected distance visual acuity, corrected distance visual acuity, and refractive outcomes. Only the study with the longest follow-up time was included if the data was used in several studies. In addition, nonoriginal studies, including reviews, letters, and comments, were excluded.

### 2.3. Data Extraction and Quality Assessment

Two authors independently extracted data according to a predefined information sheet. The information, including the first author's name, publication year, study location, sample size, patients' characteristics, the number of cases, and controls, as well as outcome data, were extracted from each individual study. The Cochrane risk assessments tool was used to evaluate the quality of studies [9], including random sequence generation, allocation concealment, blinding of participants and personnel, blinding of outcome assessment, incomplete outcome data, selective reporting, and other biases.

### 2.4. Statistic Analysis

The outcomes and complications of FLACS versus CPCS were performed using RevMan 5.2. The pooled weighted mean differences (WMD) with 95% confidence intervals (CIs) were calculated to evaluate the differences between the two techniques. The potential heterogeneity across studies was evaluated by Cochran's Q and *I*^2^ statistics [10]. *P* < 0.05 and/or *I*^2^ > 50% was considered statistically significant. The random effect model was used in case of significant heterogeneity. Otherwise, the fixed-effect model was used. Sensitivity analysis was performed through omitting one study each time to evaluate the stability of the meta-analysis.

## 3. Results

### 3.1. Study Selection and Characteristic

**Figure 1:**
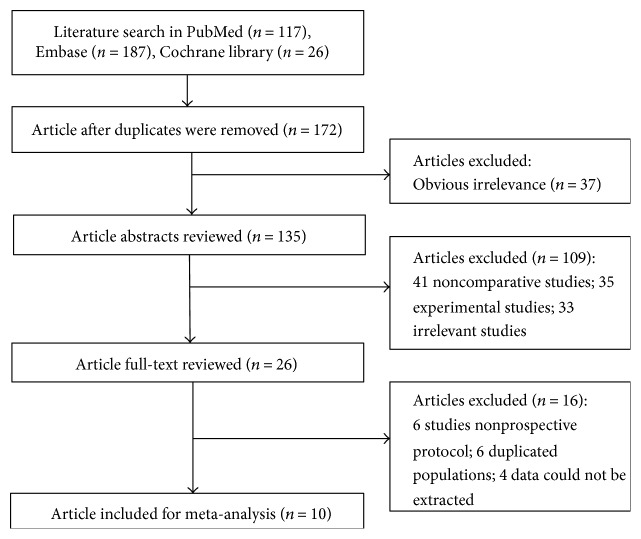
Flow chart of literature search and study selection.

The study selection process was illustrated in Figure 1. The search strategy originally yielded a total of 330 articles (117 articles from Embase database, 187 from PubMed database, and 26 from Cochrane library). After eliminating duplicated articles, 172 articles were included. Thirty-seven articles were removed after reviewing article titles. After reviewing the article abstracts, 109 articles were excluded, including 41 noncomparative studies, 35 experimental studies, and 33 noncataract patients. After reviewing the full-text of the 26 remaining articles, 10 prospective studies were finally selected for this meta-analysis ([5–7, 11–17], Table 1), including 8 from European countries, 1 from China [16], and 1 from Tasmania [7].

### 3.2. Evaluation of Risk of Bias

**Figure 2:**
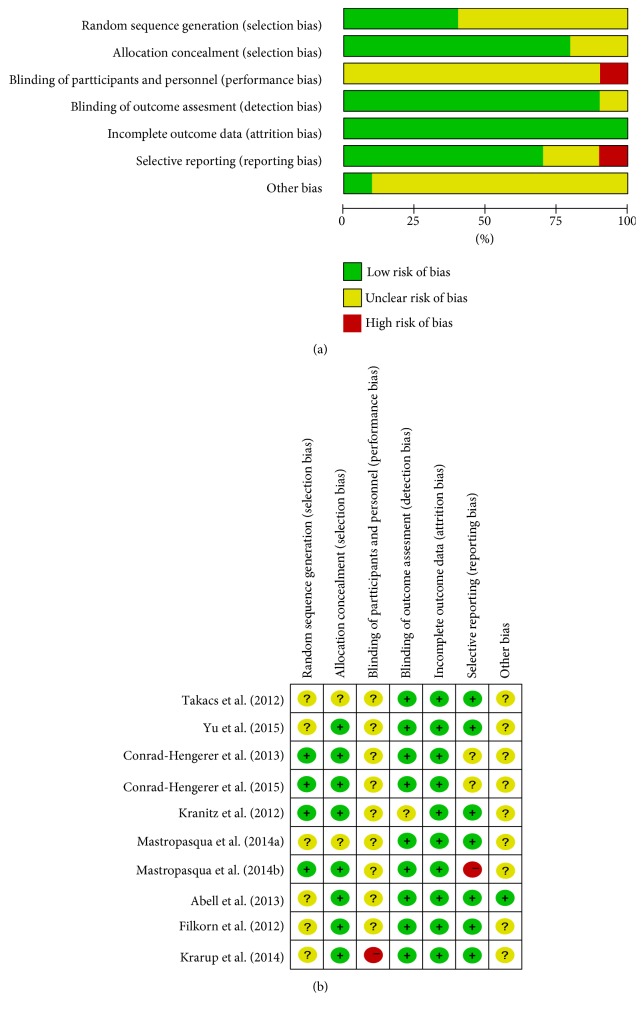
Risk of bias evaluation. (a) Risk of bias graph. (b) Risk of bias summary.

The risk of bias was shown in Figure 2. Generation of the randomization sequence was adequate in four trials. Blinding design was described in none of the enrolled studies. One study had a high risk of selective reporting because the author did not report all the outcome data that were described in the protocol.

### 3.3. Meta-Analysis of Operation Outcomes

**Figure 3:**
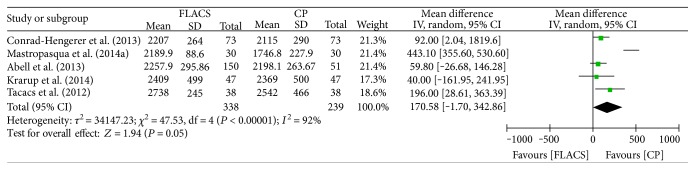
Forest plots displaying the effect of femtosecond laser-assisted cataract surgery (FLACS) versus conventional phacoemulsification cataract surgery (CPCS) on corneal endothelial cell.

Five studies evaluated corneal endothelial cell count as an outcome measure [5–7, 11, 17]. Evidence of heterogeneity was observed across these trials (*I*^2^ = 92%, *P* < 0.00001), and a random effect model was applied to pool the results (Figure 3). Corneal endothelial cell counts after CPCS was significantly less than FLACS (MD = 190.58, 95% CI: −1.70–342.86, *P* = 0.05). Heterogeneity was reduced to 0% after the study by Mastropasqua et al. was removed [6], and the results showed that FLACS significantly reduced corneal endothelial cell counts compared to CPCS (MD = 86.11, 95% CI: 29.99–142.23, *P* = 0.003).

**Figure 4:**
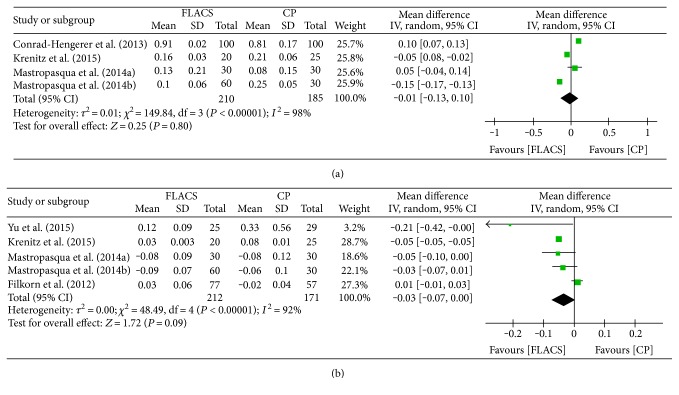
Forest plots displaying the effect of femtosecond laser-assisted cataract surgery (FLACS) versus conventional phacoemulsification cataract surgery (CPCS) on visual acuity. (a) Uncorrected distance visual acuity. (b) Corrected distance visual acuity.

Visual acuity was compared in 6 studies [6, 12–16], of which 4 evaluated uncorrected distance visual acuity (UDVA) and 5 compared corrected distance visual acuity (CDVA). As shown in Figure 4(a), significant heterogeneity was observed among the studies evaluating UDVA (*I*^2^ = 98%, *P* < 0.00001) and no significant difference in UDVA was observed between FLACS and CPCS (MD = −0.01, 95% CI: −0.13–0.10, *P* = 0.80).  Figure 4(b) shows significant difference of postoperative CDVA using random effects model (MD = −0.03, 95% CI: 0.07–0.00, *P* = 0.09). Heterogeneity was reduced to 8% after the study by Filkorn et al. was omitted [12], and the result was not inversed when we removed other studies.

**Figure 5:**
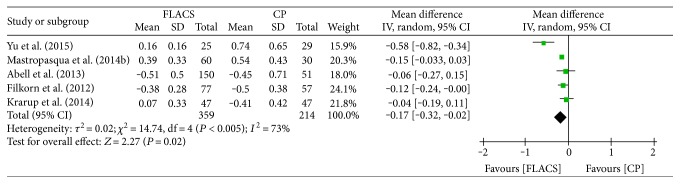
Forest plots displaying the effect of femtosecond laser-assisted cataract surgery (FLACS) versus conventional phacoemulsification cataract surgery (CPCS) on refractive outcome.

Mean absolute error (MAE) was adopted to assess refractive outcomes in 5 articles [7, 12, 14, 16, 17]. As shown in Figure 5, significant heterogeneity was calculated among studies evaluating refractive outcomes (*I*^2^ = 73%, *P* = 0.05) and FLACS that showed MAE in FLACS group was significantly lower than that in CP group (MD = −0.17, 95% CI: −0.32–0.02, *P* = 0.02). Heterogeneity was reduced to 8% after the study by Yu et al. was omitted [16], and the result was not inversed when we removed other studies.

### 3.4. Postoperative Complications

Among the enrolled studies, 3 described the occurrence of complications associated with the two surgeries [5, 15, 16]. Complications, including elevated intraocular pressure and macular edema, were most commonly reported. Moreover, the study by Yu et al. reported that pupil miosis occurred in 1 eye and mild subconjunctival hemorrhage occurred in 5 eyes after FLACS [16]. In total, 3 patients in FLACS group and 7 CPCS group developed macular edema. Additionally, elevated intraocular pressure was observed in 4 patients after FLACS and 7 patients after CPCS. Overall, the incidence of elevated intraocular pressure and macular edema during FLACS and CPCS was similar.

## 4. Discussion

A previous study by Chen et al. has suggested that FLACS is superior to CPCS for the reduction of mean phaco energy and effective phacoemulsification time [18]. Ultrasound energy introduced by conventional phacoemulsification may damage surrounding structures, resulting in endothelial cell loss [19, 20]. Therefore, a reduction in ultrasound phacoemulsification may markedly reduce postoperative corneal endothelial cell loss [21, 22]. In this study, FLACS was not superior to CPCS on postoperative corneal endothelial cell loss (MD = 197.82, 95% CI: 2.66~392.97, *P* = 0.05). However, when the study by Mastropasqua et al. was removed [6], corneal endothelial cell loss after FLACS was significantly lower than that in CPCS (MD = 86.11, 95% CI: 29.99–142.23, *P* = 0.003). Nevertheless, the conclusion needs to be validated by future studies. The positioning of intraocular lens is the most critical factor influencing the refractive outcomes [15]. Previous studies have suggested earlier stabilization of refraction after FLACS [23]. Consistently, our study found that refractive stability was significantly improved after FLACS (MD = −0.21, 95% CI: −0.39~0.03, *P* = 0.02).

It has been previously suggested that FLACS has a lower complication rate compared with CPCS [24]. In this study, we found that elevated intraocular pressure and macular edema were the most commonly reported complications. Although our study revealed a slightly lower number of cases with elevated intraocular pressure and macular edema, the incidence of elevated intraocular pressure and macular edema during FLACS and CPCS was similar. FLACS might slightly reduce the occurrence of macular edema when compared with CPCS, which might be associated with a slightly higher risk for elevated intraocular pressure probably due to the heat generated by ultrasound during the procedure [3].

Notably, significant heterogeneity was observed among the enrolled studies, which would weaken the strength of our conclusions. The heterogeneity might be attributed to various regional background, follow-up period, and surgical expertise. For instance, heterogeneity in refractive outcomes was significantly reduced (0%) after we removed the Chinese patients in Yu et al. [16]. Further, we performed sensitivity analysis, and the nonreversed results confirmed that the conclusion of our meta-analysis was reliable.

There are several limitations in the current study. First, the number of selected studies and patients are relatively small, which might affect the accuracy of our results. Furthermore, although sensitive analysis has shown the stability of our conclusions, significant heterogeneities were detected in initial analysis. Finally, given that surgical expertise could not be adjusted rigorously, our conclusions need to be verified by a study in a much larger population.

In summary, our meta-analysis found that FLACS could significantly improve refractive outcomes. Although FLACS was not superior to CPCS in reducing corneal endothelial cell loss, there was a trend towards reduced corneal endothelial cell loss after FLACS. Elevated intraocular pressure and macular edema were the most commonly developed complications. The incidence of these complications was similar after FLACS and CPCS. Our study provided evidence supporting higher treatment efficacy of FLACS based on refractive stability and corneal endothelial cell protection. However, further study is needed to validate our findings.

## Figures and Tables

**Table 1:** Characteristics of studies included in the meta-analysis.

Study	Area	Follow-up	Design	*n*, age (experimental)	*n*, age (control)	Main outcomes	Complications
Takacs et al. [11]	Hungary	4 weeks	Prospective case-control	38, 65.8 (12.4) ys	38, 66.9 (11.0) ys	Central corneal thickness, endothelial cell count	NA
Kranitz et al. [13]	Hungary	48 weeks	Prospective, randomized study	20, 68.2 (10.8) ys	25, 63.6 (13.7) ys	Refractive outcomes	NA
Filkorn et al. [12]	Germany	1 month	Prospective case-control	77, 65.2 (12.6) ys	57, 64.4 (12.4) ys	CDVA and refractive	NA
Abell et al. [7]	Tasmania	3 weeks	Prospective, consecutive, single-surgeon case-control	150,72.8 (10.5) ys	51, 71.8 (10.8) ys	Corneal endothelial cell loss, CDVA, intraocular pressure, and refractive outcomes	NA
Conrad-Hengerer et al. [5]	Germany	3 months	Randomized intraindividual cohort study	73, 70.9 ys	73, 70.9 ys	Endothelial cell counts and corneal thickness	FLACS: EIP in 3 eyes; macular edema in 2 eyes CPCS: EIP in 2 eyes, macular edema in 5 eyes
Mastropasqua et al. [6]	Italy	24 weeks	Prospective randomized study	30, 70.2 (2.9) ys	30, 70.5 (3.2) ys	UDVA, CDVA, and corneal endothelial cell counts	NA
Mastropasqua et al. [14]	Italy	24 weeks	Prospective randomized clinical study	60, 69.3 (3.2) ys	30, 69.1 (3.9) ys	UDVA, CDVA, refractive outcomes	NA
Krarup et al. [17]	Denmark	3 months	Prospective case-control	47	47	UDVA, CDVA, central corneal endothelialcell count	NA
Conrad-Hengerer et al. [15]	Germany	6 months	Prospective randomized intraindividual cohort study	100, 71.6 ys	100, 71.6 ys	Manifest refraction, corrected distance visual acuity, and anterior chamber depth	FLACS: 1 eye developed macular edema; EIP in 3 eyes; CPCS: 2 eyes developed macular edema, 1 eye developed subclinical macular edema, EIP in 2 eyes
Yu et al. [16]	China	3 months	Prospective study	25, 62.3 (11.6) ys	29, 56.5 (16.6) ys	CDVA, refractive outcomes	FLACS: pupil miosis in 1 eye, mild subconjunctival hemorrhage in 5 eyes, EIP in 1 eye; CPCS: posterior capsular opacification in 2 eyes

ys: years; FLACS: femtosecond laser-assisted cataract surgery; UDVA: uncorrected distance visual acuity; CDVA: corrected distance visual acuity; EIP: elevated intraocular pressure; NA: not available.
